# Supplementary material for: The Influence of Reduced Graphene Oxide on the Texture and Chemistry of N,S-Doped Porous Carbon. Implications for Electrocatalytic and Energy Storage Applications
Source: Nanomaterials (Basel). 2023 Aug 18;13(16):2364. doi: 10.3390/nano13162364 (PMC10460025; doi:10.3390/nano13162364)
Supplement: Supplementary file 1 [file nanomaterials-13-02364-s001.zip › nanomaterials-2522345-supplementary.pdf]

SUPPLEMENTARY MATERIAL

INFLUENCE OF REDUCED GRAPHENE OXIDE ON THE TEXTURE AND CHEMISTRY OF N, S-DOPED POROUS CARBON. IMPLICATIONS FOR ELECTROCATALYTIC AND ENERGY STORAGE APPLICATIONS

Samantha K. Samaniego Andrade[1], Shiva Shankar Lakshmi [2], István Bakos[2], Szilvia Klébert[2], Robert Kun[2,3], Miklós Mohai[2], Balázs Nagy[4], Krisztina László[1*]

[1] Department of Physical Chemistry and Materials Science, Faculty of Chemical Technology and Biotechnology, Budapest University of Technology and Economics, 1521 Budapest, Hungary

[2] Institute of Materials and Environmental Chemistry, Research Centre for Natural Sciences, Magyar tudósok körútja 2., Budapest, H-1117, Hungary

[3] Department of Chemical and Environmental Process Engineering, Faculty of Chemical Technology and Biotechnology, Budapest University of Technology and Economics, 1521 Budapest, Hungary

[4] H-ion Research, Development and Innovation Ltd., 1121 Budapest Konkoly-Thege út 29-33

*Corresponding author: laszlo.krisztina@vbk.bme.hu

FIGURES

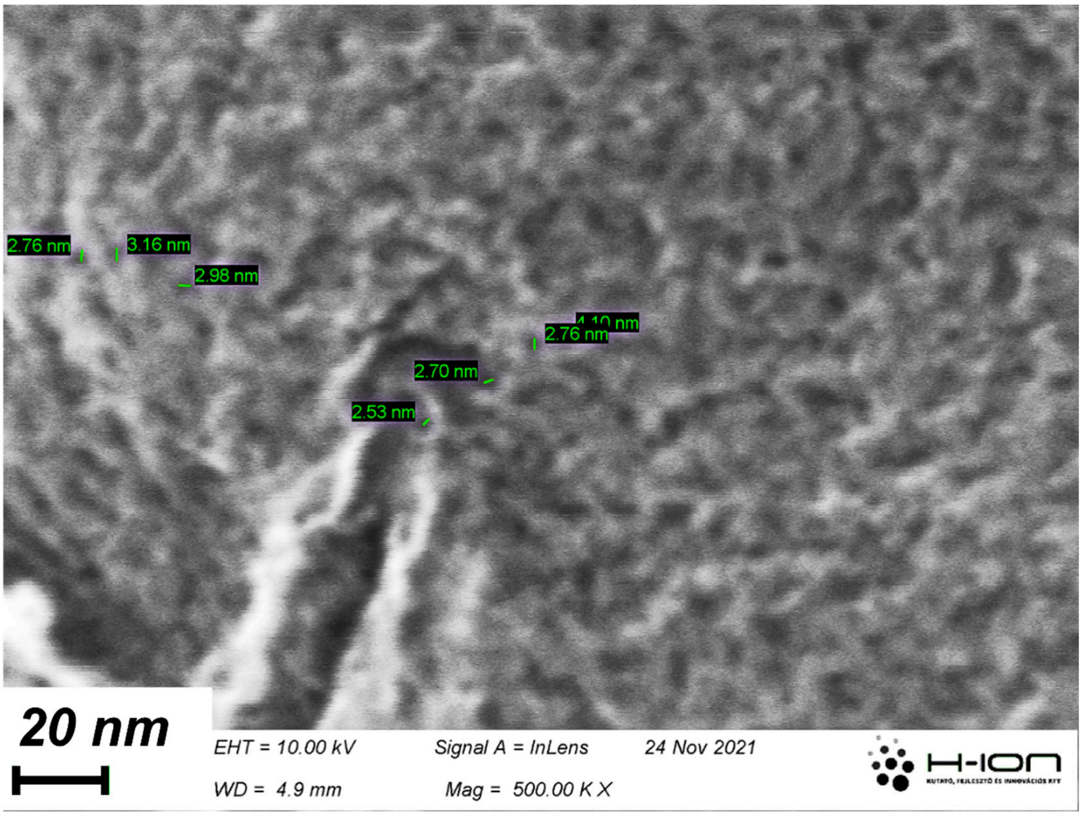

Figure S1 SEM image of the CA matrix showing nanopores of width 2–3 nm

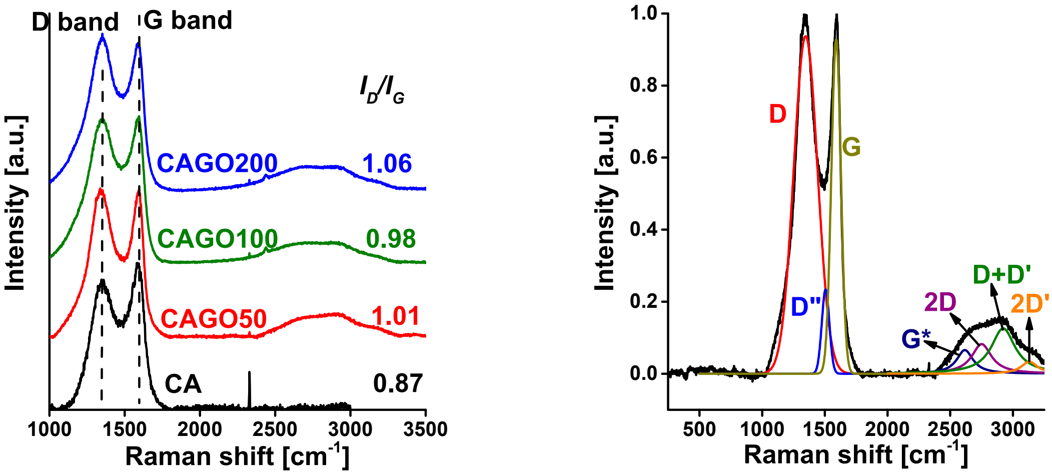

a

b

Figure S2 Raman spectra of the annealed carbon samples (a); deconvoluted Raman spectrum of CAGO50 (b). The peaks in the first order region are assigned as G: graphitic band, related to the vibrations of the sp^2 building blocks; D: defect band, related to the structural disorder; D’: disordered graphitic lattices; D”: amorphous carbon [89, 90, 91], and in the second order region as 2D: structural order; G*, D+D’, and 2D’ [92]. See also Tables S1-S2.

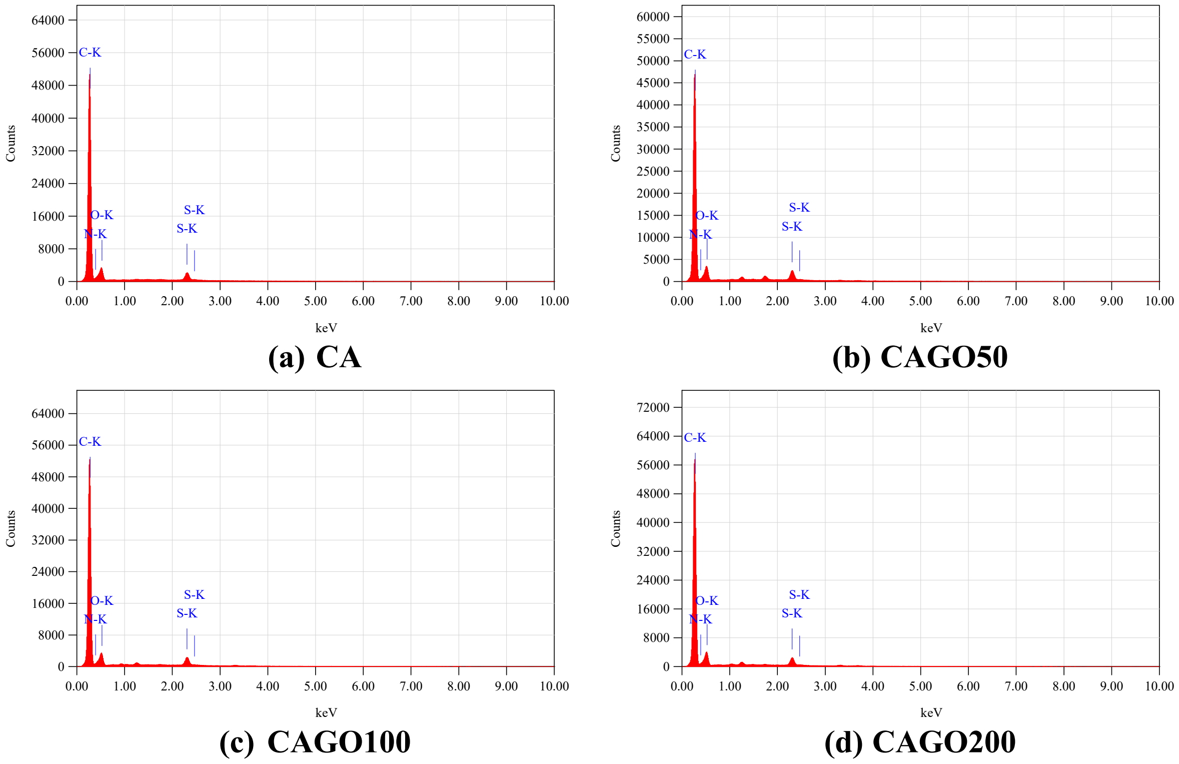

Figure S3 SEM/EDS spectra of the samples (at magnification x100)

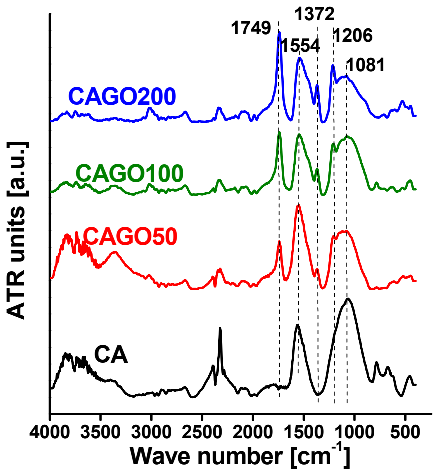

Figure S4 FTIR spectra of the carbon samples. See also Table S4

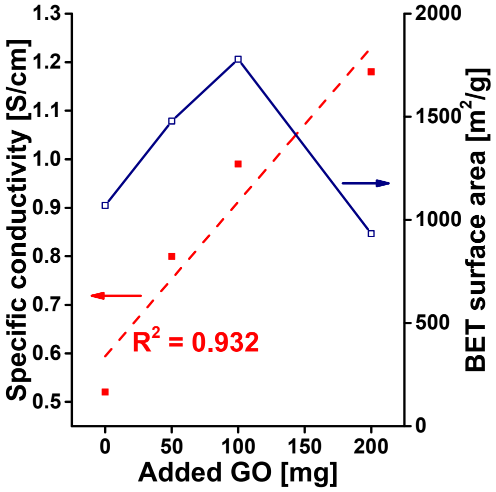

Figure S5 Influence of added GO on the specific conductivity and apparent surface area

The **effect of the electrode loading** was studied with the sample CAGO50 (**Figure S6**). The mass specific CV signals (**Figure S6b**) almost coincide, i.e., the active surface area increases proportionally with increasing coverage of the glassy carbon surface only up to 100 μg/cm^2 loading. At 400 μg/cm^2 the deformed shape of the mass specific current density curve implies that the deep carbon layer influences the transfer mechanism. The excessive amount of carbon covering the GC surface turns into a 3D coating and obstructs the electron diffusion, as corroborated by **Figure S6c**: reducing the polarization speed from 50 mV/s to 10 mV/s leads to a more ideal CV curve even for this high loading, as this allows more time for the electrolyte to reach the equilibrium state.

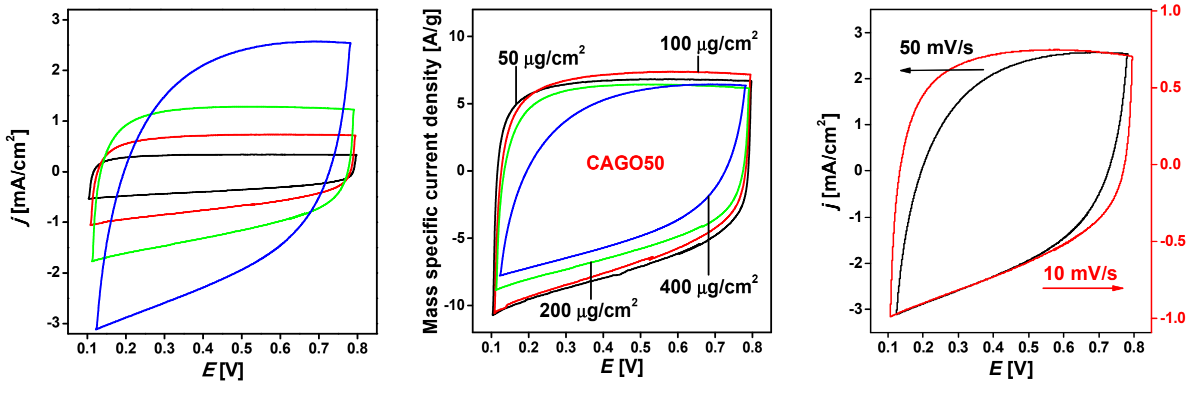

Figure S6 Effect of loading on the electrode (a) Cyclic voltammogram of CAGO50 covered GC electrode in 0.1 M KOH at various loadings: 50 (black), 100 (red), 200 (green), 400 (blue) μg/cm^2. Sweep rate: 50 mV/s. (b) Mass specific cyclic voltammogram of CAGO50 covered electrode in 0.1 M KOH; (c) Loading: 400 μg/cm^2, sweep rate 50 mV/s (black) and 10 mV/s (red).

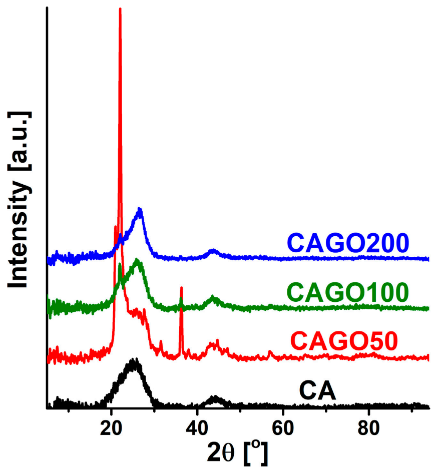

Figure S7 XRD diffractograms of CA and rGO doped carbon samples. Normalized to peak at 2θ ≈ 25–26 position

TABLES

Table S1 Yield of the synthesis steps (%)

Sample	Carbonization	Acid washing	Annealing
CA	10	53	67
CAGO50	9	63	35
CAGO100	12	66	37
CAGO200	15	68	45

Table S2 Raman shifts from the deconvoluted spectra (Figure S2b) [cm^{-1}]*

	1st order region					2nd order region			
	D	D"	G	D'	I_D/I_G	G*	2D	D+D'	2D'
[128]	1350	1506	1580	1600		2450	2700	2900	3100
CA	1353	1527	1597	1619	1.39				
CAGO50	1347	1506	1590		1.01	2617	2750	2922	3131
CAGO100	1358	1516	1593		1.11	2498	2699	2917	3088
CAGO200	1355	1514	1590		1.12	2495	2689	2920	3131

* Raman spectra were deconvoluted into Gaussian peaks using the peak analyzer function in Origin software. G: graphitic band, related to the vibrations of the sp^2 building blocks; D: defect band, related to the structural disorder; D': disordered graphitic lattices; D": amorphous carbon [80, 81, 82], 2D: structural order; G*, D+D', and 2D' [92].

Table S3 Selected data deduced from the deconvoluted Raman spectra (Figure S2b)

Sample	Δ* [cm^{-1}]	I_G [a.u.]	Δ/I_G	$I_{D"}$ [a.u.]
CA	78	0.61	127	0.43
CAGO50	89	0.93	96	0.23
CAGO100	90	0.82	110	0.18
CAGO200	92	0.84	109	0.16

* Δ is the full width of the deconvoluted G band at half maximum (FWHM)

Table S4 Ratio of the various carbon species based on FTIR spectra (Figure S4)

**Sample**	**C=O/C=C**	**OH/C=C**
CA	0.16	0.05
CAGO50	0.57	0.25
CAGO100	1.03	0.41
CAGO200	1.38	0.58
